# Sentinel Surveillance of COVID-19: The Importance of Epidemiologic Concepts and Reasoning

**DOI:** 10.2188/jea.JE20240200

**Published:** 2025-02-05

**Authors:** Yuzo Arima, Takuri Takahashi, Ayu Kasamatsu, Takeshi Arashiro, Yusuke Kobayashi, Miyako Otsuka, Osamu Takahara, Reiko Shimbashi, Katsuhiro Komase, Taro Kamigaki, Motoi Suzuki

**Affiliations:** 1 Center for Surveillance, Immunization, and Epidemiologic Research, National Institute of Infectious Diseases, Tokyo, Japan; 2 Department of Pathology, National Institute of Infectious Diseases, Tokyo, Japan; 3 Center for Field Epidemic Intelligence, Research and Professional Development, National Institute of Infectious Diseases, Tokyo, Japan

**Keywords:** surveillance, bias, restriction, SARS-CoV-2, influenza

A year has passed since surveillance for coronavirus disease 2019 (COVID-19) in Japan changed from a notifiable to a sentinel-based scheme, under the National Epidemiological Surveillance of Infectious Diseases program.^1^^,^^2^ From May 2023, COVID-19 no longer required a dedicated system or notification of all detected cases. Instead, using the pre-existing sentinel surveillance platform, the reporting sites, form, and processes all became aligned with those of seasonal influenza,^1^^–^^3^ representing a critical turning point for COVID-19 surveillance. Earlier in the pandemic, with low population immunity and no or limited vaccines/antivirals, surveillance focused on containment and mitigation/control (entailing contact-tracing and isolation/quarantine), requiring ascertainment of all cases.^4^^,^^5^ However, by spring 2023, with the highly incident and milder Omicron variant well-established, the objective of surveillance had largely transitioned to trend monitoring. While not suitable earlier (when COVID-19 was less incident), systematically tracking trends for common infectious diseases is usually best addressed through sentinel surveillance.^4^^,^^6^^–^^8^ In addition to its efficiency, the implemented sentinel scheme has distinct advantages that reduce biases and improve interpretation. Here, we outline these key concepts, reminding readers that epidemiologic reasoning plays a valuable role in public health practice.

A major feature of COVID-19 surveillance during the early years of the pandemic was the high *intensity* of surveillance, which made comparisons with other surveilled diseases, notably influenza, challenging. As a similarly acute, pandemic-prone respiratory viral disease responsible for high morbidity and mortality,^4^^,^^6^^,^^9^ comparisons with influenza were deemed important, but with caveats regarding comparability.^10^^,^^11^ Differential ascertainment is a well-acknowledged bias in surveillance^12^^,^^13^ and epidemiologic studies,^14^^,^^15^ but by making the reporting sites and structure identical (with point-of-care tests accepted for both), comparisons between the two diseases became more valid. Specifically, 1) the underlying population (case-patients for both disease groups captured from the same source population that seek care at the same facilities, as in a test-negative design^14^) and 2) the methods of data collection and collation (biases due to differential information retrieval reduced), became equal for the two. As with epidemiologic practice, sampling comparators arising from the same source—using the same data collection and measurement process—reduces potential biases.^15^ This alignment allows for more valid comparisons between the reported influenza and COVID-19 cases—the improved comparability aids clinicians with situational awareness, clinical diagnoses, and understanding of their respective burden, and, the aligned design facilitates communication and comprehension by using the same metric (ie, number of reported cases per sentinel site).^1^^,^^2^^,^^7^

The “Global Epidemiological Surveillance Standards for Influenza” states that “small amounts of good quality data will be more useful than large amounts of poor quality data”.^6^ To this end, data from designated sentinel sites that consistently and continuously report verified data based on established criteria are deemed more valid and interpretable than all-case reporting from all physicians.^4^^,^^6^^–^^8^^,^^13^^,^^15^ Restricting to sentinel sites not only helps with the aforementioned relative comparisons to influenza, but also improves temporal and geographic comparability of COVID-19 activity. Specifically, reporting from sentinel sites is less prone to biases associated with changes or differences in awareness/motivation (eg, clinicians forgetting or losing motivation to report)^8^^,^^15^ or testing schemes (eg, including asymptomatic cases detected through screening),^16^ which had indeed occurred at different times and places during the pandemic.^5^^,^^13^^,^^17^

As some biases can be difficult to control for in the analysis stage, mitigating potential biases by design^15^ is also an important method in surveillance. Based on the aligned, restricted strategy, we feed back the reported sentinel COVID-19 and influenza data weekly at https://www.niid.go.jp/niid/ja/idwr.html (by week, prefecture, and case counts stratified by age group and sex), in line with the World Health Organization’s integrated sentinel approach for both diseases.^4^ Given their unpredictable and epidemic-prone nature,^4^^–^^6^ ongoing monitoring of both diseases in a comparable and consistent manner is crucial for public health.
